# Progress in the study of mefloquine as an antibiotic adjuvant for combination bacterial inhibition treatment

**DOI:** 10.3389/fcimb.2024.1470891

**Published:** 2024-11-28

**Authors:** Xiaofang Liang, Zhihong Liu, Yulin Wang, Yu Zhang, Wenbo Deng, Qianqian Liu, Zhangping Lu, Keke Li, Yanbing Chang, Lianhua Wei

**Affiliations:** ^1^ School of Public Health, Gansu University of Chinese Medicine, Lanzhou, Gansu, China; ^2^ Department of Clinical Laboratory, Gansu Provincial Hospital, Lanzhou, Gansu, China; ^3^ Ningxia Medical University, Yinchuan, Ningxia, China

**Keywords:** mefloquine, antibiotic adjuvant, multidrug-resistant bacteria, derivatives, bacterial cell membrane, biofilm

## Abstract

Antimicrobial resistance is among the greatest threats to public health globally, and drug repurposing strategies may be advantageous to addressing this problem. Mefloquine, a drug traditionally used to treat malaria, has emerged as a promising antibiotic adjuvant, due to its ability to enhance the effectiveness of conventional antibiotics against resistant bacterial strains. In this paper, we first outline the enhancement properties of mefloquine and its mechanisms of action as an adjuvant antibiotic against multidrug-resistant bacteria. Mefloquine exhibits synergistic bacteriostatic effects when combined with colistin, β-lactams, antituberculosis drugs, quinolones, and linezolid. Potential mechanisms underlying its synergistic effects include inhibition of antibiotic efflux, disruption of bacterial cell membrane integrity, and disturbance of biofilm formation. In addition, we explore the bacteriostatic effects of several mefloquine derivatives against *Mycobacterium tuberculosis* and some fungi. Further, we summarize the findings of recent studies on other aspects of mefloquine activity, including its antiviral and antitumor effects. Finally, the advantages and challenges of mefloquine use as an antibiotic adjuvant in combination with antibiotics for bacterial inhibition are discussed. Overall, mefloquine shows excellent potential as an antibiotic adjuvant therapy against multidrug-resistant bacteria and is a promising candidate for combination therapy; however, further studies are needed to fully elucidate its mechanism of action and address the challenges associated with its clinical application.

## 1 Introduction

Antibiotics, among the most important medical discoveries of the 20th century, remain the main anti-infective drugs and have saved countless lives, while improving quality of life for humanity broadly (Cook and Wright, 2022). Despite continuous development and advances in medical science, genetic changes and the widespread and irrational use of antibiotics in healthcare, animal husbandry, and agriculture have led to the emergence and rapid spread of antimicrobial resistance (AMR) and multidrug-resistant (MDR) bacteria, with disastrous consequences for health and the economy. According to global data, 4.95 million deaths in 2019 were linked to bacterial AMR, of which 1.27 million deaths were directly attributable to bacterial AMR (Murray et al., 2022). It is projected that the annual number of deaths from bacterial AMR by 2050 (10 million) will even exceed that attributable to cancer (8.2 million) (Laxminarayan et al., 2013). Although the number of newly approved antimicrobial drugs has slightly increased over the past five years, it remains insufficient to address the growing problem of AMR. The primary impediment to progress is that identifying new drug targets and novel antibacterial compounds through whole-cell phenotypic, target-based, or gene identification studies is lengthy, costly, and not commercially profitable, which has led to gradual withdrawal of large pharmaceutical companies from the antibiotic market (Koh Jing Jie et al., 2022; Vila et al., 2020). The search for antibiotic adjuvants to existing antimicrobial drugs offers a productive and valuable approach to this problem. In most cases, adjuvants do not kill bacteria directly. However, when combined with existing antibiotics, they can increase the antibiotic’s antimicrobial efficiency by increasing the accumulation of the antibiotic in the bacterial cell or interfering with the bacterial defense system. Therefore, it is essential to tap into novel antibiotic adjuvants that can enhance the activity of existing antibiotics and extend their lifespan.

Drug combinations usually include two active compounds or one antibiotic and one non-antibiotic adjuvant molecule (Meric-Bernstam et al., 2023), where the latter can overcome the redundancy of safe but ineffective or obsolete antibiotics. An example of a clinically approved combination therapy is β-lactam with a β-lactamase inhibitor, which has played a significant role in treating infections with drug-resistant strains of bacteria. Combination therapies are common and critical in many other areas of medicine. Examples include cancer treatment (Prager et al., 2023; van der Heijden et al., 2023) or artemisinin-based combination therapy for malaria (Sutanto et al., 2023; Yeka et al., 2005). An ideal drug combination should simultaneously fulfill the following three conditions: (1) synergistic effect, mutually enhancing drug efficacy and therapeutic effect can be achieved using the lowest dose of the drug; (2) reduction of bacterial mutation rate and slowing down the development of drug resistance; and (3) even at high concentrations, no toxicity to the host cell (Sharma et al., 2021).

In March 1990, mefloquine (MFL) was recommended by the Centers for Disease Control for malaria chemoprophylaxis in areas where *Plasmodium falciparum* is endemic and was first marketed in U.S. pharmacies in May 1990 by Roche under the trade name Lariam (Kozarsky and Eaton, 1993). In recent years, MFL has increasingly been studied as a potential adjuvant for antibiotics against MDR bacteria. In this paper, we provide the first overview of the synergistic effects and potential mechanisms of MFL activity when used in combination with conventional antibiotics for treatment of MDR bacteria. Further, we summarize the antibacterial potential of MFL derivatives, as well as possible MFL applications in other areas of medical research. Finally, we discuss the favorable pharmacological properties of MFL, in terms of bacterial inhibition, and the associated shortcomings and challenges. The aim of this review is to provide new ideas to inform future deployment of synergistic combinations of MFL and antibiotics to address the antibiotic resistance crisis.

## 2 MFL and related research

MFL is a synthetic 4-quinoline-methanol derivative that is structurally very similar to the first potent antimalarial drug, quinine (Kucharski et al., 2022). MFL is effective against all strains of malaria known to infect humans and has an essential role in prevention and in unimmunized individuals, as well as in treating malaria caused by MDR *Plasmodium falciparum* (Mairet-Khedim et al., 2023). In November 2020, the World Health Organization (WHO) released a 10-year (2000–2019) surveillance report on antimalarial drug efficacy, resistance, and response (World Health Organization, 2020). The report recommends six artemisinin-based combination therapies (ACTs) as first- and second-line treatments for *Plasmodium falciparum*, including artemisinin derivatives (artesunate, artemether, or dihydroartemisinin); artesunate-MFL is used as one of these ACTs.

Recently, an increasing number of studies have shown that MFL is an effective antibiotic adjuvant that enhances the susceptibility of various drug-resistant bacteria to a wide range of antibiotics. Additionally, research into its medical use in other areas has revealed potential for clinical applications (**Table 1**).

**Table 1:** Applications of MFL.

Category	Application	Notes and clarifications	References
Antiviral	JC Virus	Inhibition of JC virus DNA replication in the brain of immunocompromised individuals and blocking the development of progressive multifocal leukoencephalopathy	(Brickelmaier et al., 2009; Hirayama et al., 2012; Shin et al., 2014)
	Feline calicivirus	Inhibition of feline calicivirus replication and cytopathic effects	(McDonagh et al., 2015)
	Severe acute respiratory syndrome coronavirus 2 (SARSCoV-2)	Inhibits viral entry into target cells and has *in vitro* activity against SARSCoV-2 attached to target cells	(Jan et al., 2021; Sacramento et al., 2022)
	Human coronaviruses (HCoV), 229E, and OC43	*In vitro* resistance activity against HCoV, 229E, and OC43	(Persoons et al., 2021)
	Prion	*In vitro* anti-Ruan virus activity and inhibits the formation of abnormal protease-resistant prion protein (PrP-res) in cells	(Kocisko and Caughey, 2006)
Antitumor	Gastric cancer	Inhibits gastric cancer cell proliferation and induces apoptosis by inhibiting the PI3K/Akt/mTOR pathway	(Liu Y. et al., 2016)
	Liver cancer	Selective inhibition of the proliferation and self-renewal of CD133^+^ HepG2 cells by targeting the β-catenin pathway	(Li et al., 2018)
	Malignant melanoma	MFL causes melanoma cell death at low micromolar concentrations, even in the presence of BRAF kinase inhibitor-resistance and brain metastases	(Jandova et al., 2022)
	Breast cancer	Anticancer effects of MFL on both hormone receptor-positive and -negative breast cancer cell lines	(Sharma et al., 2012)
	Cervical cancer	Impairment of mitochondrial function and inhibition of the mTOR pathway induces apoptosis in multiple cervical cancer cell lines	(Li et al., 2017)
	Colorectal cancer	Inhibits nuclear factor kappa B signaling and induces apoptosis in colorectal cancer cells	(Xu et al., 2018)
		Blocking mitochondrial autophagic degradation and inducing apoptosis in colorectal cancer stem cells by inhibiting RAB5/7, LAMP1/2, and PINK1/PARKIN in tumor cells	(Takeda et al., 2019)
	Esophageal squamous cell carcinoma	Inhibition of esophageal squamous cell carcinoma tumor growth by induction of mitochondrial autophagy	(Xie et al., 2020)
	Glioblastoma	MFL acts as a dual inhibitor of glioblastoma angiogenesis and glioblastoma via disrupting lysosomal function	(Wan et al., 2021)
	Prostate cancer	Induction of prostate cancer cell death by mediating G1 cell cycle arrest and cyclin D1 accumulation through p21 upregulation in PC3 cells	(Yan et al., 2013)
	Myeloid leukemia	MFL selectively augments the effects of BCR-ABL tyrosine kinase inhibitors in chronic myeloid leukemia stem/progenitor cells by inducing lysosomal dysfunction	(Lam Yi et al., 2019)
		MFL selectively kills acute myeloid leukemia cells and progenitor cells by disrupting lysosomes	(Sukhai et al., 2013)
	Melanoma and lung cancer	MFL induces tumor ferroptosis via IFN-γ-STAT1-IRF1-LPCAT3, enhancing the efficacy of anti-programmed cell death 1 (PD-1) immunotherapy	(Tao et al., 2024)
Inhibition of multiple membrane channels	Cardiac potassium channels KvLQT1/minK	MFL is an antagonist of the cardiac potassium channel, KvLQT1/minK, and slows its activation	(Kang et al., 2001)
	Chloride channels	MFL effectively blocks volume-regulated and calcium-activated chloride channels	(Maertens et al., 2000)
	Cx36 and Cx50 gap junction channels	Blockade of the gap junction proteins, Cx36 and Cx50, was instrumental in determining the physiological roles of these junction protein isoforms	(Cruikshank et al., 2004)
	ATP-sensitive K-channels	Inhibits β-cell ATP-sensitive K-channels and stimulates insulin secretion	(Gribble et al., 2000)
Other applications	MFL effectively inhibits NLRP3 inflammasome-mediated systemic inflammation and attenuates nerve damage		(Jiang et al., 2023)
	MFL ameliorates pulmonary fibrosis by inhibiting the macrophage KCNH2/Jak2/Stat3 signaling pathway		(Zhou et al., 2024)
	MFL can be used to prevent many complications following malaria treatment, such as thrombotic thrombocytopenic purpura and acute fatty liver disease		(Grieco et al., 1999; Stracher et al., 1994)
	MFL increases vertebral cancellous bone formation and mass in a sclerostin-independent manner in aged animals and prevents the effects of aging on bone strength		(Pacheco-Costa et al., 2018)
	MFL induces mast cell apoptosis through a secretory granule-mediated pathway		(Lampinen et al., 2022; Paivandy et al., 2014)

## 3 MFL structure and metabolism

**Figure 1:**
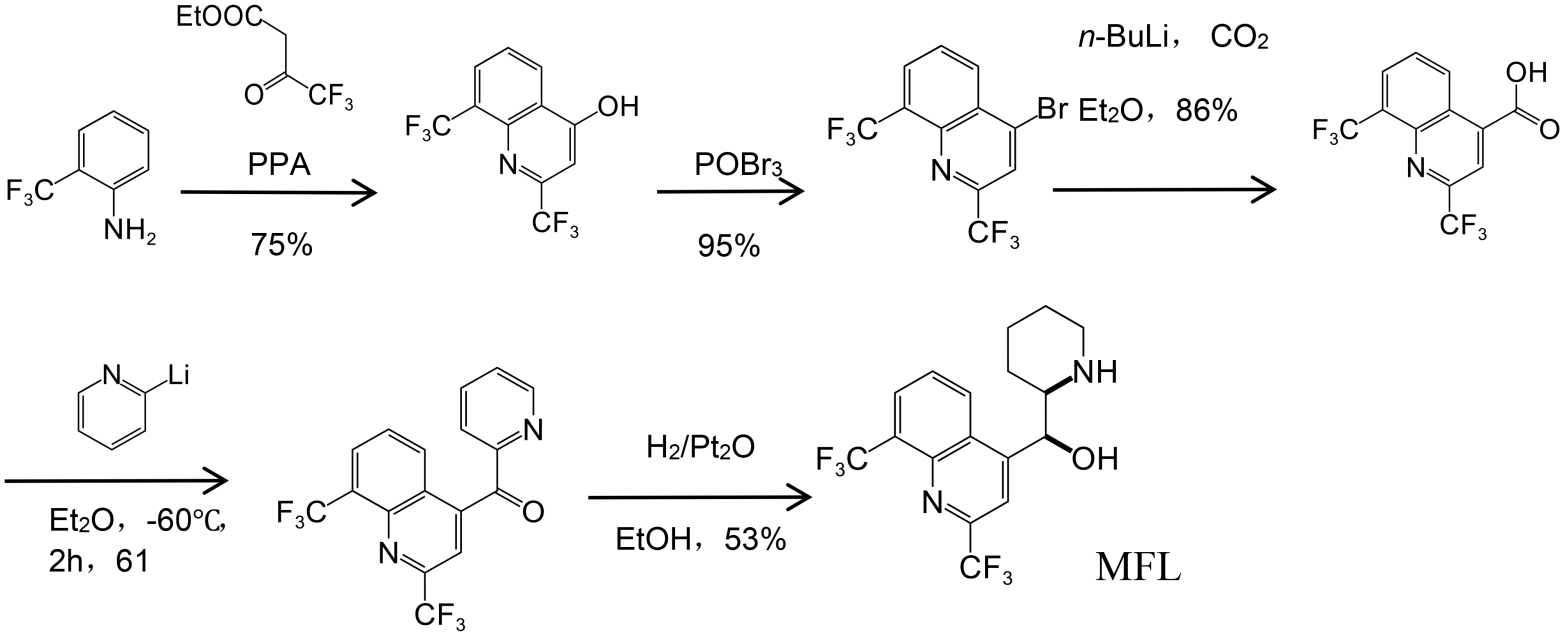
Synthesis of MFL (Kucharski et al., 2022).

Lutz et al. first documented MFL synthesis in the 1970s (Lutz et al., 1971), and it was also briefly summarized by Kucharski et al. (2022) (**Figure 1**). MFL is a chiral compound with two asymmetric centers and exists in two racemic forms (erythro and threo), each of which comprises a pair of optical isomers; i.e., (±)-erythro-enantiomers and (±)-threo-epimers. When the term MFL is used in clinical settings, it is often referring to the erythro enantiomer (erythro-isomer racemic mixture) (Kucharski et al., 2022).

After the entry of MFL into the body, approximately 75%–80% is absorbed, with peak drug concentrations occurring between 7 and 24 h after administration (Karbwang and White, 1990). In the plasma, MFL is primarily bound to proteins in large amounts and has a high affinity for lipids, allowing it to reach very high intracellular concentrations (Wernsdorfer et al., 2013). MFL has a long half-life, and studies in mice have shown that it can be administered every three days to treat disseminated *Mycobacterium avium* infections (Bermudez et al., 2003) and weekly for treating malaria in humans (Schlagenhauf et al., 2010). MFL is extensively metabolized, primarily in the liver, by CYP3A4, to produce the pharmacologically inactive form, carboxy MFL, which is finally excreted from the body in the bile via the enterohepatic circulation (Piedade et al., 2015).

The main disadvantage of MFL is the occurrence of dose-related neuropsychiatric adverse effects, including abnormal dreaming, anxiety, nausea, and dizziness (the last two being the most common symptoms), with women and individuals with a lower body mass index (BMI) being more affected by the side effects (Toovey, 2009). Due to the side effects of MFL, a doctor’s prescription is required in countries such as Australia (Administration (TGA), T.G, 2022), Canada (Government of Canada, 2013) and the United Kingdom (Medicines and Healthcare Products Regulatory Agency, 2024). The United States Food and Drug Administration (2017) and the European Medicines Agency (Schlagenhauf et al., 2015) are also cautious about the use of MFL because its safety is not fully proven, and studies on the mechanism of action of MFL neurotoxicity still need to be better understood. A limited number of studies have shown that enantiomer (-)-erythro- MFL binds to adenosine receptors and cholinesterase in the central nervous system, regulates neurotransmitter release, disrupts intracellular homeostasis to produce oxidative stress, and impairs the function of voltage-dependent calcium channels and gap junctional intercellular communication are thought to be responsible for neuropsychiatric symptoms, whereas (+)- erythro- MFL enantiomer does not bind tightly to adenosine receptors in the brain (Schmidt et al., 2012). Therefore, selective synthesis and use of (+)-erythro- MFL rather than racemates could result in a better risk-benefit assessment. Müller et al. (2013) applied residual dipolar coupling (RDC)-enhanced NMR spectroscopy in combination with optical rotatory dispersion (ORD) and circular dichroism(CD) spectroscopy, determined the (+)-erythro- MFL absolute configuration to be (11S, 12R), and the absolute configuration of (-)-erythro- MFL to be (11R, 12S). Selective synthesis of (+)-erythro- MFL enantiomers could provide safer antimalarial drugs. Although MFL has the side effects of neuropsychiatric adverse effects, considering the wide range of potential applications of MFL in antibacterial, antitumor, and antiviral (**Table 1**), it is worthwhile for researchers to conduct neurotoxic mechanism of action studies, such as new tools based on transcriptomic and proteomic analysis to gain new insights into MFL-induced neurotoxicity and to identify and provide potential early biomarkers or molecular targets that may be involved in its adverse neurological effects for further investigation.

## 4 The synergistic effects of MFL with antibiotics

Antibiotic adjuvants can include compounds or herbal products with known pharmacology or toxicology that do not have direct bactericidal activity, but may enhance the antibacterial efficacy of an antibiotic through various mechanisms (e.g., blocking resistance, enhancing intracellular antibiotic accumulation, complementing the bactericidal pathway, inhibiting signaling and modulation pathways, or augmenting the host response to bacterial infection) (Sharma et al., 2012). Synergism is commonly measured using the fractional inhibitory concentration index (FICI); a criterion used to assess synergism, additivity, or antagonism between two drugs (Coates et al., 2020). The FICI is calculated using the checkerboard method, which involves generating 96 different concentrations containing gradient dilutions of combinations of two drugs and observing their effects on the growth of microorganisms, to determine the optimal concentration that produces the most potent interaction.

The formula for calculating FICI is as follows:


$$\begin{array}{c} \text{FICI }=\frac{\text{MIC of A in combination with B}}{\text{MIC of A alone}}\\ \;\;+\frac{\text{MIC of B in combination with A}}{\text{MIC of B alone}} \end{array}$$

Where MIC indicates ‘minimum inhibitory concentration’; and FICI values are interpreted as follows: FICI ≤ 0.5, synergistic; FICI > 0.5–4.0, additive; FICI > 4.0, antagonistic.

We used the above approach to evaluate the effectiveness of MFL as an adjuvant in combination with other antibiotics for the treatment of clinically prevalent MDR bacteria, including gram-negative bacteria (e.g., *Pseudomonas aeruginosa*, *Escherichia coli*, and *Klebsiella pneumoniae*), gram-positive bacteria [e.g., methicillin-resistant *Staphylococcus aureus*), and mycobacteria (*Mycobacterium tuberculosis* and *M. avium* complexes (MACs)].

### 4.1 Gram-negative bacteria

Gram-negative drug-resistant bacteria are the most dangerous group of MDR bacteria. Their unique bacterial membrane structure makes them more resistant than gram-positive bacteria and causes significantly higher rates of detection, morbidity, and mortality worldwide. The WHO updated the list of “priority pathogens” in need of antibiotics in May 2024 (World Health Organization, 2024), and gram-negative bacteria remain the main critical priority among bacteria, including carbapenem-resistant *Acinetobacter baumannii*, third-generation cephalosporin-resistant and carbapenem-resistant *Enterobacteriaceae*, and rifampicin-resistant *M. tuberculosis.* Compared with the 2017 list, carbapenem-resistant *P. aeruginosa* (CRPA) was downgraded from critical to high priority; however, the WHO emphasizes that investment in research and development and other prevention and control strategies for CRPA remains important, given its high burden in some regions. Gram-negative resistant bacteria are resistant to carbapenems, and resistance to the “last resort” treatment, colistin, is also rising, emphasizing the increased need for new therapeutic measures to control the threat to humans posed by infections with these organisms.

#### 4.1.1 P. aeruginosa

*P. aeruginosa* is an opportunistic pathogen closely associated with many acute and chronic diseases, and infection with this bacterium is a significant cause of high morbidity and mortality in patients with acquired pneumonia (Fernández-Barat et al., 2017), chronic obstructive pulmonary disease (Garcia-Nuñez et al., 2017), and cystic fibrosis (Rossi et al., 2021). In addition, biofilm formation leads to resistance of intrafilm bacteria to antibiotics, complicating treatment of these diseases and causing recurrent infections. Colistin is considered the last resort treatment for infections caused by carbapenem-resistant bacterial strains (Liu et al., 2022); however, use of colistin has been abandoned due to its severe side effects, such as nephrotoxicity and neurotoxicity (Nang et al., 2021; Tsuji et al., 2019); therefore, it is essential to plan therapeutic strategies, including combination treatments with nonantibiotic drugs, to reduce these side effects by lowering colistin concentrations.

One study showed that, in combination with colistin, MFL had excellent synergistic antibacterial activity against colistin-resistant *P. aeruginosa* (**Table 2** and **Supplementary Table S1**) (Zhang et al., 2021). Drug susceptibility and checkerboard assays of colistin-resistant *P. aeruginosa* clinical isolates revealed MIC values of colistin against the tested bacteria of 4–128 μg/mL, and when MFL (4–32 μg/mL) was used in combination a colistin, it reduced the MIC values of colistin to 0.125–2.000 μg/mL, with FICI values of 0.047–0.188 (much lower than 0.5). Hence, MFL reduced the MIC values of colistin by 8- to 64-fold, and significant changes in susceptibility to colistin were detected in all strains, from those with resistant to those with sensitive phenotypes. In an *in vivo* infection model, combining 1 μg/mL colistin and 64 μg/mL MFL for 168 h resulted in 100% survival of bumblebee larvae (**Table 2** and **Supplementary Table S1**) (Zhang et al., 2021). In addition, a combination of the immunomodulator, AS101, with MFL had ex vivo and *in vivo* inhibitory effects on CRPA. Evaluation of the *in vivo* therapeutic effects of AS101 combined with MFL showed that it effectively reduced the bacterial load in organs including the liver, kidney, and spleen in a mouse model infected with CRPA (Li et al., 2023).

**Table 2:** Synergistic effects of MFL and antibiotics on bacteria.

Bacterial strain		No. of strains	Antibiotic			FICI	References
			Name	MIC alone (μg/ml)	MIC in combination (μg/ml)		
*P. aeruginosa*		8	Colistin	4–128	0.125–2	0.04–0.19	(Zhang et al., 2021)
*E. coli*	*Bla NDM* plasmid	2	Colistin	0.24	0.03-0.06	0.38-0.5	(Hu and Coates, 2021)
	*mcr-1* plasmid	13	Colistin	2-4	0.01–0.5	0.13–0.5	
	ESBL-producing	42	Colistin	0.08–8	0.01–0.25	0.19–0.5	
*K. pneumoniae*	*Bla NDM* plasmid	4	Colistin	0.48–2.08	0.06–0.13	0.19–0.25	
	ESBL-producing	46	Colistin	0.1–10.25	0.01–0.5	0.13–0.5	
*S. aureus*	MRSA	2	Colistin	128-512	32-128	0.5	(Podoll et al., 2021)
	MSSA	2	Colistin	0.25	0.06	0.5	
*M. tuberculosis*	H37Rv	1	INH	0.2	0.1	0.5	(Dos Santos et al., 2021)
		1	PYR	100	3.12	0.3	
		1	OFX	1.25	0.62	0.5	
	T3609	1	INH	0.5	0.015	0.03	
		1	GAT	0.62	0.31	0.5	
		1	MOX	1.25	0.62	0.5	
		1	SPR	1.25	0.62	0.5	
	T113	1	CPX	0.62	0.31	0.5	
		1	LVX	0.62	0.31	0.5	
		1	OFX	1.25	0.62	0.5	

The general font is directly compared to the tabular information in the references. MIC, Minimum Inhibitory Concentrations; MFL, mefloquine; INH, isoniazid; PYR, pyrazinamide; OFX, ofloxacin; GAT, gatifloxacin; MOX, moxifloxacin; SPR, sparfloxacin; CPX, ciprofloxacin; LVX, levofloxacin; *P. aeruginosa, Pseudomonas aeruginosa; E. coli, Escherichia coli; K. pneumoniae, Klebsiella pneumoniae;* MRSA, methicillin-resistant *Staphylococcus aureus;* MSSA, methicillin-susceptible *Staphylococcus aureus; M. tuberculosis, Mycobacterium tuberculosis.*

#### 4.1.2 Carbapenem-resistant *Enterobacteriaceae*

The worldwide emergence of carbapenem-resistant *Enterobacteriaceae* (CRE) remains the most pressing category of AMR threat, as these organisms are incredibly resistant to most antibiotics (Hsu et al., 2017). The main resistance mechanisms in CRE are carbapenemase-producing enzymes, high production of ultra-broad-spectrum β-lactamases (ESBLs), or altered membrane permeability, due to mutations in exocytosis pumps or porins (Potter et al., 2016). Common carbapenemases include class A (KPC), class B (IMP, NDM, VIM), and class D (OXA-48) (Han et al., 2020). As CRE is rapidly sweeping the globe, many countries have turned to the use of colistin, which has become an important therapeutic option for treating infections caused by CRE (D’Onofrio et al., 2020; Rojas et al., 2017); however, discovery of the MCR-1 gene (Liu Y.-Y. et al., 2016) and the increased use of colistin has led to increasingly significant CRE resistance to this cationic short peptide. Therefore, it is crucial to improve and maintain the effectiveness of colistin against CRE.

In combination with colistin, MFL has synergistic antimicrobial activity against *Enterobacteriaceae* bacteria harboring *NDM-1* or *mcr-1* genes or producing ESBL (**Table 2** and **Supplementary Table S1**) (Hu and Coates, 2021). The synergistic activity of colistin with MFL was tested against *E. coli* and *K. pneumoniae* containing the *bla_NDM_* plasmid, and combination with MFL reduced the MIC of colistin from 0.25–2.00 μg/mL to 0.06–0.13μg/mL, representing a 4- to 16-fold reduction; the FICI index was ≤ 0.5 in both cases. Further, synergistic activity of this combination was tested against *E. coli* containing the *mcr-1* plasmid, and the results showed that the MIC of *E. coli* to colistin was reduced from 2.00–4.00 μg/mL to 0.02–0.50 μg/mL, representing an 8- to 256-fold reduction; however, the efficacy of combination treatment with MFL and colistin was observed to vary between strains for ESBL-producing CRE. Testing of 48 ESBL-producing *E. coli* strains and 47 K*. pneumoniae* strains showed that the MIC of colistin reduced from 0.125–8.00 μg/mL to 0.01–0.50 μg/mL, representing a range of 4- to 256-fold reduction. Only additive effects were observed in six of the *E. coli* strains and one of the *K. pneumoniae* strains (FICI index values, 0.5–2), while synergistic effects were observed in the remaining strains (FICI index ≤ 0.5)

In mouse peritoneal infection models of *NDM-1*-positive *K. pneumoniae* BAA2470 and *mcr-1*-positive *E. coli* Af45, a decrease in peritoneal bacterial counts was observed in mice after just 4 h treatment with a combination of 20 mg/kg MFL and 20 mg/kg colistin. Importantly MFL enhances the antimicrobial effects of colistin, reduces the dose of colistin required, decreases host toxicity, and maintains maximum therapeutic efficacy (Hu and Coates, 2021).

### 4.2 Gram-positive bacteria

The WHO has designated gram-positive vancomycin-resistant *Enterococcus* and methicillin-resistant *S. aureus* (MRSA) as high-priority categories requiring new antimicrobial drug therapies (World Health Organization, 2024). There is an urgent clinical need for new antimicrobial agents or effective treatment strategies to combat the therapeutic challenge of drug-resistant gram-positive cocci infections.

MRSA infections occur globally and can invade hospitals, healthcare facilities, and communities, as well as being found in livestock (Lakhundi and Zhang, 2018). MFL can disrupt the phospholipid membranes of *S. aureus*, alter membrane fluidity, and enhance the susceptibility of MRSA or methicillin-susceptible *S. aureus* (MSSA) to the β-lactam antibiotic, **o**xacillin (Podoll et al., 2021). When MFL was added at a subinhibitory concentration (1/4 MIC), it reduced the MIC values of MSSA and MRSA to **o**xacillin by 4-fold (**Table 2** and **Supplementary Table S1**). Hence, application of MFL as an antibiotic adjuvant offers a promising approach to the treatment of MRSA-induced infections (Podoll et al., 2021).

### 4.3 Mycobacteria

The emergence of drug-resistant mycobacteria, including *M. tuberculosis* and nontuberculous mycobacteria (NTM), poses a growing threat globally. In 2022, MDR tuberculosis (MDR-TB) was the largest airborne drug-resistant epidemic worldwide. If left unaddressed, it is predicted that MDR-TB will cost the global economy approximately $17 trillion by 2050 (Dheda et al., 2024). Diseases caused by NTM infection have also increased worldwide, resulting in an urgent clinical need to develop new effective antimycobacterial drugs (Dahl et al., 2022).

#### 4.3.1 M. tuberculosis

The WHO classifies rifampicin-resistant *M. tuberculosis* as a critical priority bacterium (World Health Organization, 2024). After humans are infected with *M. tuberculosis*, the bacterium usually lives in an acidic granulomatous environment or hypoxic phagocytic vesicles, which results in the inactivation of most antituberculosis drugs. A study (Bermudez and Meek, 2014) shows that, *in vitro*, MFL exhibits similar antimicrobial effects against *M. tuberculosis* even under hypoxic conditions as it does in the presence of oxygen, with an MIC of 8 μg/mL. Antimicrobial activity was also observed under acidic conditions (MIC = 8 μg/mL), which is important because, among antituberculosis compounds, only pyrazinamide is fully antituberculosis active under acidic conditions. MFL also showed significant antibacterial activity against the sensitive strain, H37Rv (ATCC 27294), in macrophages. Although the *in vitro* MIC of MFL against H37Rv was 8 μg/mL, antimicrobial activity was still observed when infected macrophages were treated with a MFL at a serum concentration of 4 μg/mL (Gonçalves et al., 2012) because MFL can be enriched in erythrocytes, hepatocytes, and macrophages at concentrations up to 80 times those detected in serum. This characteristic is important in acting against bacteria that can parasitize within cells and is considered desirable in any antituberculosis compound.

Treatment using MFL in combination with two first-line antituberculosis drugs, isoniazid and pyrazinamide, and six quinolones (gatifloxacin, moxifloxacin, ciprofloxacin, levofloxacin, ofloxacin, and sparfloxacin) has synergistic inhibitory effects against *M. tuberculosis* (**Table 2** and **Supplementary Table S1**) (Dos Santos et al., 2021). Combination treatment of the resistant isolate, T3609 (resistant to ofloxacin and streptomycin), with MFL and isoniazid, reduced the MIC of isoniazid from 0.500 to 0.015, a 33-fold reduction (FICI = 0.03, much lower than 0.5). Further, combined MFL with pyrazinamide to treat H37Rv resulted in an FICI value of 0.3.

The six quinolones all acted synergistically with MFL against at least one sensitive strain (H37Rv) and the two clinically resistant strains (T3609 and T113; resistant to isoniazid, rifampicin, ethambutol and ofloxacin). Gatifloxacin, moxifloxacin, and sparfloxacin in combination with MFL had synergistic effects against T3609 (FICI = 0.5), while combination of MFL with ciprofloxacin, levofloxacin, and ofloxacin had synergistic effects against the T113 isolate (FICI = 0.5). Further, ofloxacin combined with MFL had synergistic effects against both H37Rv and T113 (FICI = 0.5). Notably, no antagonistic effects were observed (**Table 2** and **Supplementary Table S1**) (Dos Santos et al., 2021).

#### 4.3.2 NTM

Among NTM species, MAC infection is a common cause of bacteremia in patients with advanced AIDS. In addition, this bacterium is inherently resistant to many commonly used antibiotics (Kumar et al., 2022; Nguyen and Daley, 2023); hence, treatment of MAC-induced lung infections is challenging.

*In vitro* bacterial inhibition assays showed that the MIC of MFL against MDR MAC was 16 μg/mL; however, significant inhibition of MAC in macrophages was observed when the extracellular concentration was ≥ 10 μg/mL. Evaluation of *in vivo* efficacy in a mouse infection model demonstrated that MFL significantly inhibited MAC, and significantly reduced the bacterial load in mouse liver and spleen at doses of 30 mg/kg three times per week or 20 mg/kg daily (Bermudez et al., 1999). Commercially available MFL is a racemic mixture containing four different isomers, including (+)-erythro-, (-)-erythro-, (+)-threo-, and (-)-threo-MFL, each of which may exhibit different biological activities. The MIC values of the four isomers against MAC are reported as 32, 32, 64, and 64 μg/mL, respectively, and *in vivo* studies have shown that (+)-erythro-MFL is most efficient in reducing the bacterial load in mice (Bermudez et al., 2012).

MFL also exhibits synergistic antimicrobial activity with linezolid, ethambutol, and moxifloxacin. In a patient with chronic lymphocytic leukemia who developed diffuse cutaneous MAC lesions, which were refractory to conventional antimicrobials due to resistance, the combination of linezolid and MFL improved the skin lesions and successfully treated refractory diffuse MAC infections (Nannini et al., 2002). In addition, in combination with ethambutol and moxifloxacin, MFL significantly reduced the liver and spleen bacterial load in a mouse infection model and effectively improved the mouse survival rate (Bermudez et al., 2004, 2003).

## 5 Antimicrobial activity of MFL derivatives against *M. tuberculosis*

**Figure 2:**
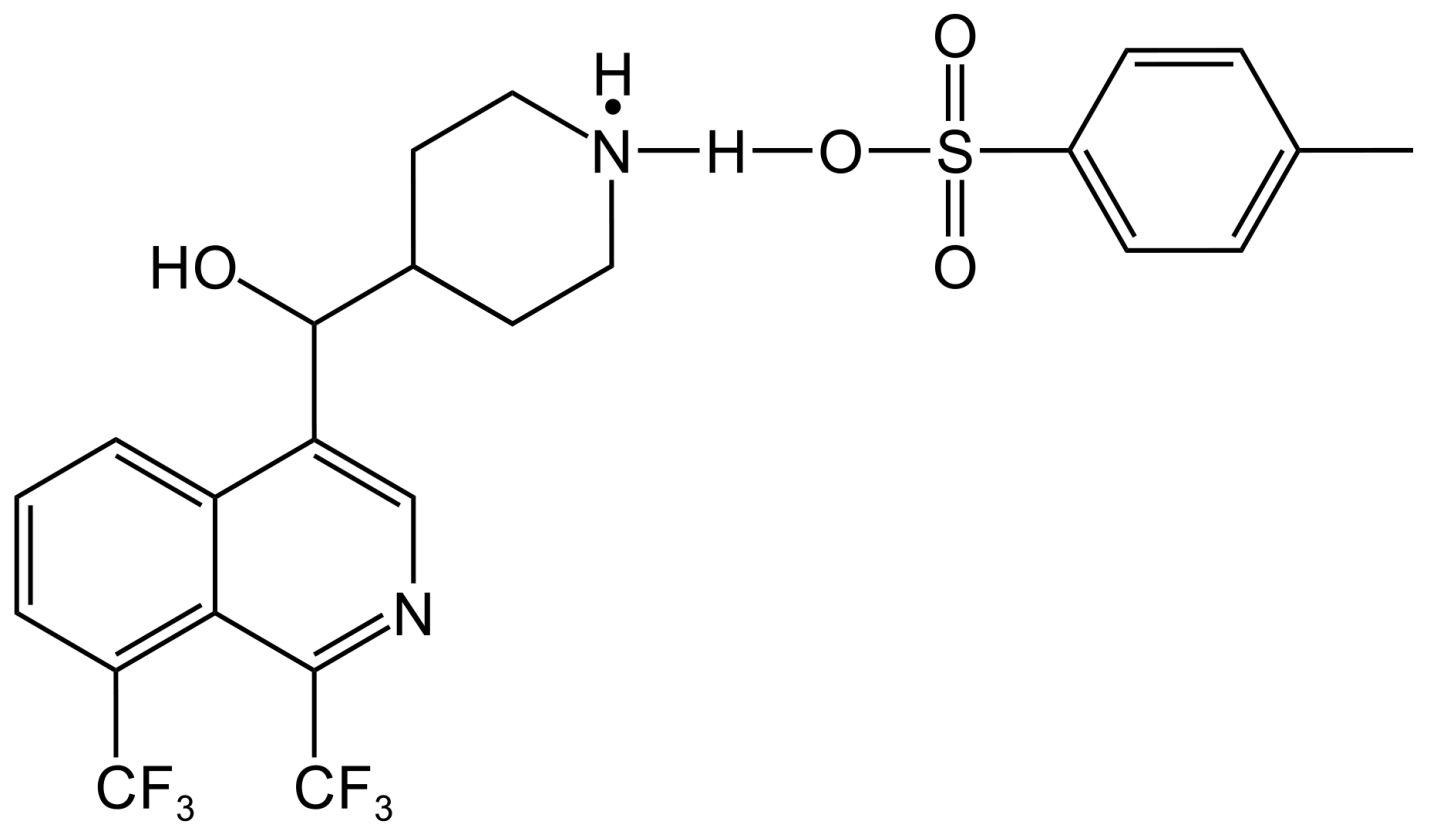
Structure of [MFLH][TsO].

Several MFL derivatives with potent antitubercular activity have been described (**Table 3**). Silva et al. (2022) used MFL hydrochloride ([MFLH][Cl]) as a raw material and complexed it with several sodium salts of organic acids to obtain eight MFL organic salts, all of which showed higher bioavailability than the raw materials. Among them, the compound [MFLH][TsO] (**Figure 2**) obtained via tosylate (TsO) was the most promising, in terms of antituberculosis effects, showing the highest activity against H37RV, with an MIC of 12.5 μg/mL, which is 1.3 times higher than that of [MFLH][Cl] (9.6 μg/mL), and was not cytotoxic to macrophages at the MIC.

**Table 3:** Antimicrobial activity of MFL derivatives against *M. tuberculosis*.

Compound	Strain	MIC (μg/mL)	References
[MFLH][TsO]	H37RV	12.5	(Silva et al., 2022)
3C	H37RV	12.5	(da Silva Araújo et al., 2019)
	SR 2571/0215	12.5	
7	H37RV	25	
	SR 2571/0215	51	
9	H37RV	25	
	SR 2571/0215	25	
1j	H37RV	2.7	(Gonçalves et al., 2010)
1e	H37RV	2.7	(Gonçalves et al., 2012)
	T113	6.2	
2a	H37RV	2.8	
	T113	6.3	
1E	Eleven clinically isolated strains of quinolone-resistant *M. tuberculosis*	0.5~4	(Rodrigues-Junior et al., 2016)
9d	H37Rv	0.06	(Mao et al., 2009)
3	Replicating *M. tuberculosis*	0.4	(Mao et al., 2010)
	Nonreplicating *M. tuberculosis*	5.3	

da Silva Araújo et al. (2019) obtained 11 new compounds by a series of reactions centered on MFL. Among them, 3C (**Figure 3A**), 7 (**Figure 3B**), and 9 (**Figure 3C**) showed the highest activity against H37RV, with MIC values of 12.5, 25, and 25 μg/mL, respectively. MIC values against the resistant strain, SR 2571/0215 (resistant to rifampicin and isoniazid), were 12.5, 51, and 25 μg/mL, respectively. The most potent inhibitory effects against resistant and sensitive *M. tuberculosis* were observed with compound 3 C.

**Figure 3:**
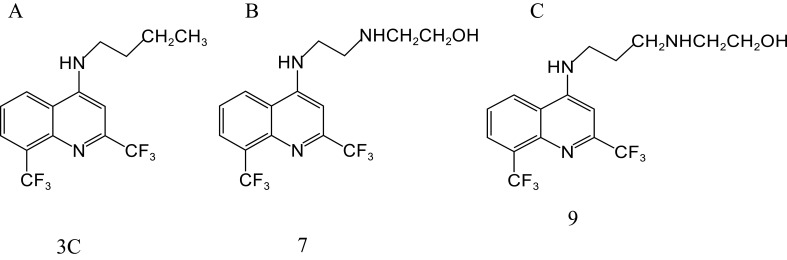
Structure of 3C **(A)**, 7 **(B)**, and 9 **(C)**.

**Figure 4:**
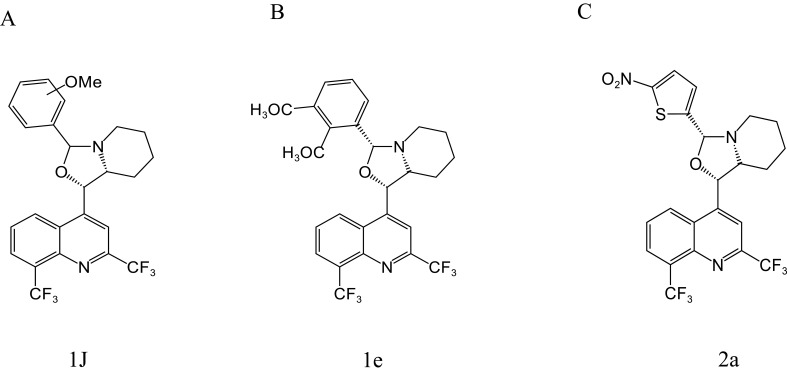
Structure of 1J **(A)**, 1e **(B)**, and 2a **(C)**.

Gonçalves et al. (2010, 2012) synthesized a series of MFL-oxazolidine derivatives in 2010 and 2012, and these new compounds were not cytotoxic, but exhibited potent antitubercular activity. Among them, compounds 1j (**Figure 4A**), 1e (**Figure 4B**), and 2a (**Figure 4C**) had MIC values of 2.7, 2.7, and 2.8 μg/mL against H37RV, which were approximately 2.6-fold higher than that of MFL, and their antimicrobial activities were even better than that of the first-line antituberculosis drug, ethambutol. Compounds 1e and 2a were also assayed for MIC against T113, and the same MIC values as those for H37RV were observed. Moreover, these compounds were not cytotoxic to mouse macrophages at concentrations close to their MIC values.

**Figure 5:**
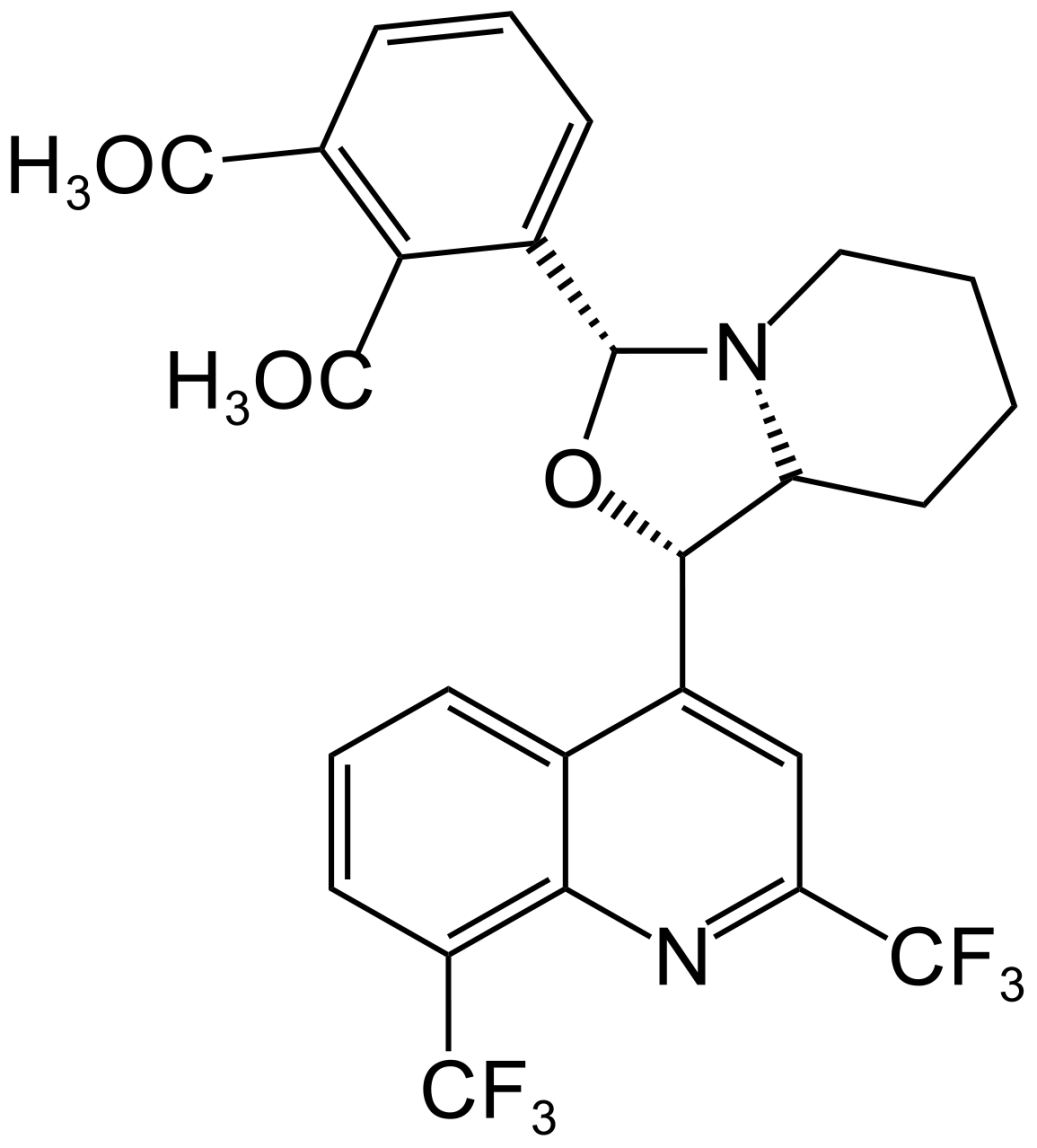
Structure of 1E.

Rodrigues-Junior et al. (2016) synthesized the MFL-oxazolidine derivative, compound 1E (**Figure 5**), which showed MIC values of 0.5–4 μg/mL against 11 clinical isolates of quinolone-resistant *M. tuberculosis*, which were lower than the MIC of MFL (8 μg/mL). In addition, 1E was effective in reducing H37Rv bacterial load in RAW 264.7 macrophages, and *in vivo* experiments demonstrated that 1E could effectively reduce the bacterial load in the lungs and spleen of an H37Rv mouse infection model.

**Figure 6:**
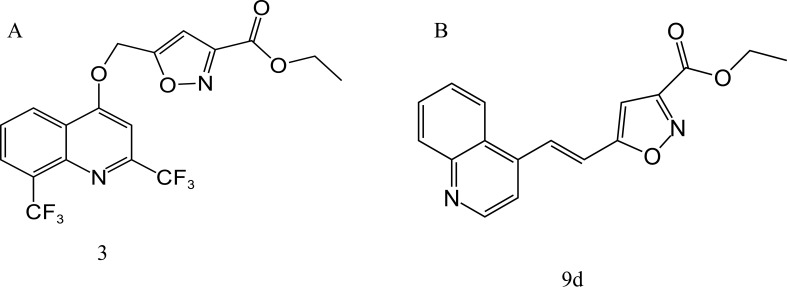
Structure of 3 **(A)** and 9d **(B)**.

In 2009 and 2010, Mao et al. (2009, 2010) synthesized a series of MFL-isoxazole carboxylates with good metabolic stability *in vitro* and *in vivo*, based on pharmacokinetic data and rational drug design principles, including compounds 3 (**Figure 6A**) and 9d (**Figure 6B**). Compound 9d had an MIC value of 0.06 μg/mL against H37Rv, which was only two-fold higher than that of RFP, and was active against H37Rv (MIC = 3.68 μg/mL) under acidic conditions (pH 6.8). Compound 3 showed good activity against both replicating and nonreplicating *M. tuberculosis*, with MIC values of 0.4 and 5.3 μg/mL, respectively. Furthermore, the corresponding acid of compound 3 showed increased antituberculosis activity in an acidic environment, and significantly reduced the number of bacteria in a macrophage infection model, suggesting that compound 3 may be active in the acidic environment produced by inflammation in the lungs of patients with tuberculosis.

## 6 Antifungal activity of MFL derivatives

Invasive fungi can cause acute CNS infections (Giuliano et al., 2023). MFL and its derivatives cross the blood-brain barrier, facilitating sufficiently high penetration into the CNS (Shin et al., 2014), which makes them attractive candidates for treatment of fungal meningitis.

Compared with MFL, MFL derivatives exhibit more efficient antifungal activity. MFL shows poor or no antifungal activity of against *Candida albicans*, *Cryptococcus neoformans*, or *Aspergillus fumigatus*, with MIC values of ≥ 128, 32, and > 64 μg/mL, respectively (Kunin and Ellis, 2000); however, MFL structural analogs showed efficient antimicrobial activity against these fungi.

**Figure 7:**
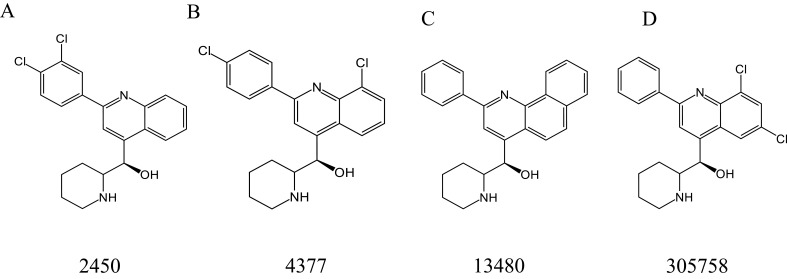
Structure of 2450 **(A)**, 4377 **(B)**, 13480 **(C)**, and 305758 **(D)**.

Montoya et al. (2020) screened four MFL derivatives with antifungal activity from the National Cancer Institute chemical library, including 2450 (**Figure 7A**), 4377 (**Figure 7B**), 13480 (**Figure 7C**), and 305758 (**Figure 7D**), of which 4377 exhibited the strongest antimicrobial activity, with MIC values of 1–4 μg/mL against all tested organisms (including *C. albicans*, *Candida glabrata*, *S. cerevisiae*, *C. neoformans*, and *A. fumigatus*), and the MIC values of the other three derivatives were also in the range of 2–8 μg/mL. In addition to direct antifungal activity, subinhibitory concentrations of MFL derivatives could reduce the expression of virulence traits, including filamentation in *C. albicans* and capsule formation/melanization in *C. neoformans* (Montoya et al., 2020).

## 7 Mechanism underlying the synergistic effects of MFL

Drugs can show synergistic relevance in various ways. The two most accepted synergy models are (1) the parallel pathway inhibition model and (2) the bioavailability model (Jia et al., 2009). In the parallel pathway inhibition model, two drugs are considered synergistic if they can simultaneously target two proteins in parallel pathways that are phenotypically critical for inhibition (Yeh et al., 2009). In the bioavailability model, two drugs are considered synergistic if one acts in a way that can contribute to increased accumulation of the concentration and availability of the other in target cells (Cokol et al., 2011). A typical example of a synergistic mechanism via the bioavailability model is when one drug enhances the intracellular concentration of another by destabilizing a drug transport barrier or inhibiting drug efflux. Although MFL may exhibit more than one mode of activity, the bioavailability model appears to be more consistent with its mechanism of action, based on the results observed when MFL acts against various bacteria with different antibiotics.

### 7.1 Inhibition of bacterial efflux pumps

**Figure 8:**
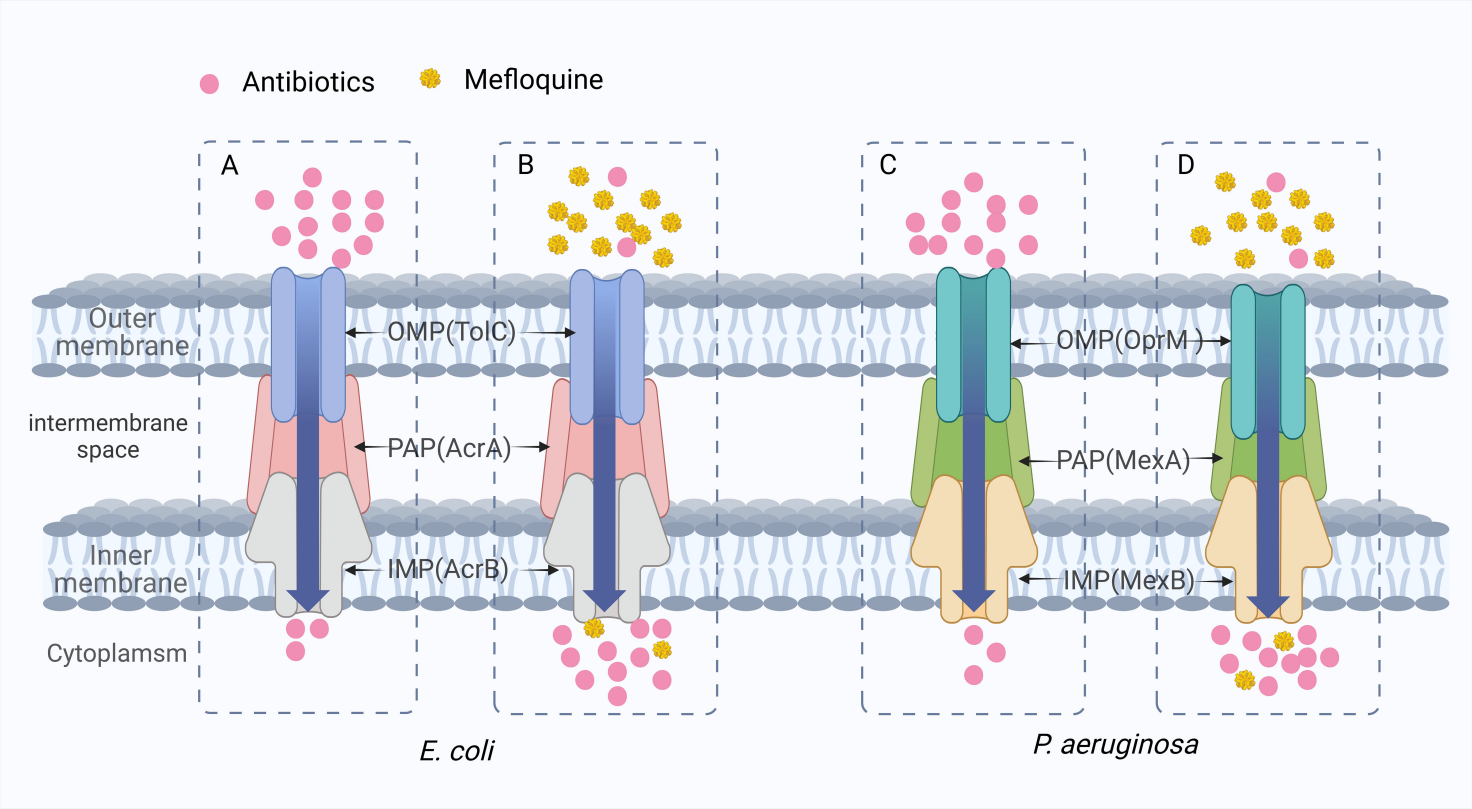
The synergistic mechanism of MFL in inhibiting antibiotic efflux. **(A, C)** When antibiotics are used alone, antibiotics are pumped out of the bacteria and drug resistance develops due to the presence of the AcrAB-TolC efflux system of *E. coli* and the MexAB-OprM efflux system of *P. aeruginosa*. **(B, D)** MFL plays a competitive role in inhibiting antibiotic efflux. In *E. coli* and *P. aeruginosa*, MFL showed greater affinity for the AcrAB-TolC efflux pump and the MexAB-OprM efflux pump. It was preferentially pumped out, reducing the efflux of other corresponding antibiotics.

It is well known that the presence of active efflux pumps on the outer membrane of bacteria is one of the main reasons for developing multidrug resistance in bacteria. Among different types of drug efflux pumps, the resistance nodulation division (RND) superfamily confers MDR to various Gram-negative bacteria species. The RND efflux pump is primarily in the trimeric form. It consists of three proteins: an inner membrane protein (IMP), an outer membrane protein (OMP), and a periplasmic adaptor protein (PAP) that connects IMP to OMP. The AcrAB-TolC efflux system from *E. coli* and the MexAB-OprM efflux system from *P. aeruginosa* are the most characteristic examples of RND efflux pumps (Puzari and Chetia, 2017). The substrates of these efflux systems are dominated by various antibiotics, which can effectively excrete various drug molecules, thereby reducing their intracellular concentrations. MFL has been identified as a potential efflux pump inhibitor as an efflux substrate for the efflux transporter AcrB of *E. coli* and as an efflux substrate for the intimal transporter MexB of *P. aeruginosa* (Schuster et al., 2022; Vidal-Aroca et al., 2009). When combined with antibiotics, it can compete with the substrate binding site of the efflux pump and inhibit the efflux of antibiotics (**Figure 8**). The reduction of efflux increases the concentration of antibiotics in bacteria and reduces the occurrence of drug resistance.

### 7.2 MFL acts on bacterial cell membranes

Bacterial membranes are involved in numerous fundamental cellular processes and often contribute directly to antibiotic resistance. Therefore, screening for drugs that can act on bacterial membranes could provide a promising method for discovery of antibiotic adjuvants (Dias and Rauter, 2019; Epand et al., 2016). When MFL acts on mycobacteria, resistant mutants are unavailable *in vitro* or *in vivo* at increased concentrations of MFL, suggesting that the mutation may be lethal or that the target of MFL may be multiple (Danelishvili et al., 2005). After exposure to subinhibitory concentrations of MFL, *M. tuberculosis* and *M. avium* up-regulate genes primarily involved in cell wall synthesis and metabolic pathways, leading to disorganization of the bacterial cell membrane, which promotes antibiotic accumulation in target cells, to exert synergistic bacteriostatic effects.

MFL treatment of M. avium resulted in the up-regulation of genes associated with the following bacterial physiological processes: lipid metabolism (*accD3*, *fadD19*, and *fadA2*), intermediary metabolism (*guaB2*), information pathways (*rpsT*, *serS*, and *infB*), regulatory proteins (*phoR*), and cellular differentiation (*Rv3661*), as well as 12 genes functionally classified as contributing to the cell wall and cellular processes, including hypothetical integral membrane proteins and transporter proteins (Danelishvili et al., 2005).

Differential gene expression analysis was also conducted using strain H37Rv that was either untreated, treated with a subinhibitory concentration of MFL, or treated with 4× MIC MFL for 24 h. Treatment with a subinhibitory concentration of MFL resulted in a total of 133 differential genes, of which 108 genes showed a > 2-fold increase in expression, and 25 genes were down-regulated; most differential genes were related to the cell wall (*mmpS4*, *arsB*, *nicT*, *amt*, *sugI*, *embB*), and anabolism pathways (*fabG2*, *pks17*, *tesb2*, *fadE24*).

**Figure 9:**
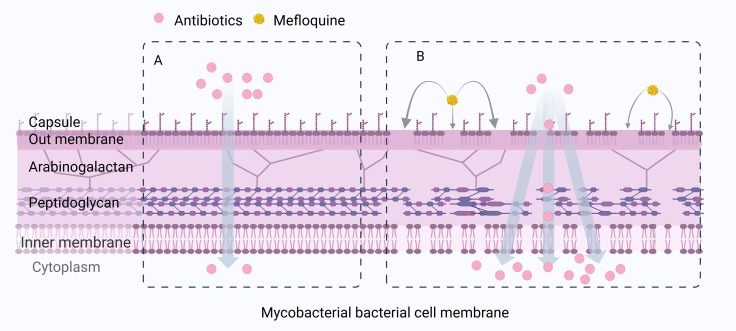
MFL can cause cell membrane disturbances, increased permeability, and decreased lipid envelopes in Mycobacterial bacteria. **(A)** When antibiotics are used alone, the cell wall of Mycobacteria is not destroyed. **(B)** When antibiotics are combined with MFL, MFL affects the integrity and permeability of the bacterial cell membrane of Mycobacteria, increases the accumulation of antibiotic concentration in the cell, and thus exerts a synergistic antibacterial effect.

Exposure to high concentrations (4× MIC) of MFL resulted in a significant stress response and expression of genes encoding heat shock proteins (*hsp*, *dnaK*, *dnaJ1*, *clpB*, *groEL2*, *groES*). MFL causes disorganization of *M. tuberculosis* bacterial cell membranes, increasing their permeability, and reducing lipid packaging, a phenomenon attributable to the “spacer” effect of the aromatic ring of MFL, due to the preferential position of MFL at the PIM2 interface, and a reduction in lipid-molecule communication, which generates a higher lateral diffusion coefficient close to the lipid interface (**Figure 9**) (Dos Santos et al., 2021).

MFL can alter the fluidity of MRSA bacterial membranes and disrupt their structural integrity. Thus, MFL promotes the intracellular permeation of the β-lactam antibiotic, oxacillin, in MRSA, increasing the intracellular concentration of the drug in bacteria (Podoll et al., 2021). These results suggest that the synergistic bacteriostatic effect associated with MFL may be attributable to its ability to affect the integrity and permeability of bacterial cell membranes, to increase the accumulation of antibiotics and their intracellular concentrations, thereby exerting synergistic bacteriostatic effects.

### 7.3 Inhibition of bacterial biofilm formation

Bacterial biofilms are multicellular three-dimensional tissues attached to the extracellular matrix secreted by bacteria on the surfaces of living or inanimate objects (Wang et al., 2016). These groups of bacteria are embedded in self-produced extracellular polymeric substances (EPSs), comprising DNA, proteins, lipids, polysaccharides, biopolymers, and divalent cations (Buzzo et al., 2021). EPSs provide a protective barrier for bacteria against invasion by antibiotics, antimicrobial agents, and host immune effects, and bacteria within biofilms are 10 to 1000 times more tolerant to antimicrobials and disinfectants than those in a planktonic state (Zhang et al., 2020). There are three main reasons for such high levels of resistance. First, nutrient and oxygen content decrease gradually from outside to inside of the biofilm, and the metabolism of deep-seated bacteria is slowed, which can contribute to bacteria retention, thereby increasing resistance to targeted antibiotics. The hypoxic environment inside the biofilm also reduces the bactericidal abilities of antibiotics. Second, the unique structure of biofilms can effectively prevent penetration by antimicrobial proteases, complement, and other macromolecules, allowing mature cells deep in the matrix more time to develop drug resistance. Third, the production and release of resistance factors by individual resistant bacteria allows horizontal transfer and uptake of resistance genes through plasmid transfer (Winans et al., 2022), where plasmid transfer is up to 700-fold more efficient among bacteria in biofilms than that in planktonic bacteria. These factors are the main mechanisms underlying the contribution of biofilms to chronic infections.

Combination treatment with MFL and colistin inhibits *P. aeruginosa* biofilm formation and eradicates pre-formed mature biofilms better than monotherapy or control group conditions (Zhang et al., 2021). MFL is a protein synthesis inhibitor that targets the 80S ribosome of *Plasmodium falciparum* to inhibit protein synthesis (Wong et al., 2017); therefore, it has been hypothesized that, in combination treatment using MFL and colistin, colistin alters *P. aeruginosa* cell membrane permeability, allowing MFL to easily enter the bacterium and inhibit biofilm protein synthesis, thus disrupting biofilm structure (Li et al., 2023). It is also possible that the combination of MFL and colistin hinders expression of the quorum sensing system, thus inhibiting biofilm formation (Zhang et al., 2021). The mechanism by which MFL inhibits biofilm formation requires further study.

## 8 Conclusion

Pathogen mutation and the spread of drug resistance are recognized as major public health problems affecting human health and food quality. In Europe and the United States alone, at least 50,000 people die annually from microbial infections (European Antimicrobial Resistance Collaborators, 2022), and the number of deaths is much higher in other parts of the world. In this context, reintroducing approved unconventional antimicrobials, or applying them as antibiotic adjuvants in combination therapies, is becoming increasingly important.

As a potential antibiotic adjuvant, MFL has little or no antimicrobial activity but can enhance the antimicrobial effect of antibiotics. Studies have shown that MFL has an antibacterial effect on *S. aureus* only at high concentrations and has no antibacterial activity against *P. aeruginosa*, *E. coli*, and *K. pneumonia*. Combined with MFL, it can reduce the resistance of many known MDR bacteria to specific antibiotics and even reverse their resistance phenotype. The antimicrobial effects of a wide range of antibiotics, including colistin, β-lactams (oxacillin), antituberculosis drugs (isoniazid, pyrazinamide and ethambutol), quinolones (gatifloxacin, moxifloxacin, ciprofloxacin, levofloxacin, levofloxacin, ofloxacin, and sparfloxacin), and oxazolidinones (linezolid), can be enhanced in combination with MFL. These effects provide new opportunities for the treatment of common clinically drug-resistant gram-negative bacteria (*P. aeruginosa*, *E. coli*, and *K. pneumoniae*), gram-positive bacteria (MRSA and MSSA), and mycobacteria (*M. tuberculosis* and MACs). The mechanisms by which MFL enhances the bactericidal activity of antibiotics include attenuating antibiotic efflux, disrupting bacterial cell wall integrity, and inhibiting biofilm formation or eradicating pre-formed mature biofilms. Despite the long-term use of MFL for treating malaria, the exact mechanism of action underlying its antimalarial activity remains unclear, and the mechanism through which MFL exerts its antimicrobial activity has not been fully elucidated. Hence, further research is warranted to provide deeper understanding of the mechanism of action of MFL, optimize its use in the clinic and infectious disease, and ensure that it can be safely and effectively applied.

In addition, various MFL derivatives with strong bactericidal effects against *M. tuberculosis* and activities against some fungi have been successfully synthesized. The known favorable pharmacological properties and novel multitarget mechanism of action of MFL provide strong support for the development and optimization of MFL scaffolds and their derivatives for the treatment of Mycobacterial and fungal infections. First, MFL has a good affinity for lipids and can reach high concentrations in serum and tissue cells after oral administration, a feature that is attractive for the treatment of intracellular *M. tuberculosis*. Second, MFL and its derivatives can penetrate the blood-brain barrier, making them promising candidates for treating infectious diseases of the CNS. Third, it has become established practice to use prophylactic strategies in patients at high risk for infectious diseases, and MFL has a long half-life and can be used in dosing regimens to prevent infection. Notably, the side effects of MFL on the CNS limit its potential use in clinical settings. However, this issue should be carefully addressed when considering the treatment of infectious diseases with MFL, as it provides a new strategy for tackling multidrug-resistant bacterial infections in the context of limited antibiotic development and a continuing rise in drug resistance. There are still limitations in the research on MFL as an antibiotic adjuvant, such as the incomplete and unsystematic design of combined antimicrobial susceptibility experiments and limited specimen size. This has hindered a comprehensive evaluation of the synergistic effect of MFL with commonly used antibiotics on susceptible, resistant, MDR, and XDR bacteria. The exact mechanism of MFL’s auxiliary function has not been fully elucidated through more profound studies, which has led to some obstacles to achieving synergistic effects *in vivo*, and solving this dilemma requires consideration of complex pharmacology and *in vivo* drug metabolism, as well as thorough toxicological evaluation, and the study and characterization of its targets in bacteria, in order to clarify the exact mechanism of MFL antibiotic adjuvant activity and better promote its clinical application.
