# Supplementary material for: Progress in the study of mefloquine as an antibiotic adjuvant for combination bacterial inhibition treatment
Source: Front Cell Infect Microbiol. 2024 Nov 28;14:1470891. doi: 10.3389/fcimb.2024.1470891 (PMC11634880; doi:10.3389/fcimb.2024.1470891)
Supplement: Supplementary file 1 [file Table1.docx]

Supplementary Material

**2 Supplementary Table S1**

Table S1 MIC values and interactions between MFL and antibiotics against bacteria

| Bacterial strain | Antibiotic | MIC alone (μg/mL) | | MIC in combination (μg/mL) | | FICI | Fold-change antibiotic MIC | Fold-change MFL MIC | Effect | References |
| --- | --- | --- | --- | --- | --- | --- | --- | --- | --- | --- |
|  |  | Antibiotic | MFL | Antibiotic | MFL |  |  |  |  |  |
| *P. aeruginosa* TL-1671 | Colistin | 32 | ＞256 | 1 | 4 | ＜0.047 | **32** | **64** | Synergy | (Zhang et al., 2021) |
| *P. aeruginosa* TL-1736 | Colistin | 16 | ＞256 | 1 | 32 | ＜0.188 | **16** | **8** | Synergy |  |
| *P. aeruginosa* TL-1744 | Colistin | 64 | ＞256 | 1 | 8 | ＜0.049 | **64** | **32** | Synergy |  |
| *P. aeruginosa* TL-2314 | Colistin | 8 | ＞256 | 0.125 | 16 | ＜0.078 | **64** | **16** | Synergy |  |
| *P. aeruginosa* TL-2917 | Colistin | 16 | ＞256 | 0.25 | 16 | ＜0.078 | **64** | **16** | Synergy |  |
| *P. aeruginosa* TL-2967 | Colistin | 4 | ＞256 | 0.5 | 16 | ＜0.188 | **8** | **16** | Synergy |  |
| *P. aeruginosa* TL-3008 | Colistin | 64 | ＞256 | 2 | 16 | ＜0.094 | **32** | **16** | Synergy |  |
| *P. aeruginosa* TL-3086 | Colistin | ＞128 | ＞256 | 2 | 16 | ＜0.078 | **64** | **16** | Synergy |  |
| *E. coli* BAA- 2469 | Colistin | 0.24 | **32** | 0.03 | 8 | 0.38 | **8** | **4** | Synergy | (Hu and Coates, 2021) |
| *E. coli* BAA- 2471 | Colistin | 0.24 | **16** | 0.06 | 4 | 0.5 | **4** | **4** | Synergy |  |
| *E. coli* AF 23 | Colistin | 2.56 | **132** | 0.02 | 16 | 0.13 | **128** | **8.25** | Synergy |  |
| *E. coli* AF 24 | Colistin | 4 | **64** | 0.25 | 8 | 0.19 | **16** | **8** | Synergy |  |
| *E. coli* AF 31 | Colistin | 4 | **32** | 0.5 | 8 | 0.38 | **8** | **4** | Synergy |  |
| *E. coli* AF 40 | Colistin | 1.92 | **32** | 0.06 | 8 | 0.28 | **32** | **4** | Synergy |  |
| *E. coli* AF 45 | Colistin | 3.84 | **64** | 0.03 | 16 | 0.26 | **128** | **4** | Synergy |  |
| *E. coli* AF 48 | Colistin | 2.56 | **32** | 0.01 | 16 | 0.5 | **256** | **2** | Synergy |  |
| *E. coli* AF 49 | Colistin | 4 | **32** | 0.25 | 8 | 0.31 | **16** | **4** | Synergy |  |
| *E. coli* CDF 1 | Colistin | 4 | **32** | 0.5 | 8 | 0.38 | **8** | **4** | Synergy |  |
| *E. coli* CDF 6 | Colistin | 4 | **64** | 0.25 | 8 | 0.19 | **16** | **8** | Synergy |  |
| *E. coli* CDF 8 | Colistin | 2.56 | **64** | 0.01 | 16 | 0.25 | **256** | **4** | Synergy |  |
| *E. coli* PS 1 | Colistin | 4 | **125** | 0.25 | 8 | 0.19 | **16** | **15** | Synergy |  |
| *E. coli* S115 | Colistin | 1.92 | **64** | 0.03 | 8 | 0.14 | **64** | **8** | Synergy |  |
| *E. coli* A1 | Colistin | 0.24 | **64** | 0.06 | 8 | 0.25 | **4** | **8** | Synergy |  |
| *E. coli* A2 | Colistin | 0.32 | **64** | 0.02 | 16 | 0.31 | **16** | **8** | Synergy |  |
| *E. coli* A3 | Colistin | 0.32 | **64** | 0.02 | 16 | 0.31 | **16** | **8** | Synergy |  |
| *E. coli* A4 | Colistin | 0.24 | **32** | 0.06 | 8 | 0.5 | **4** | **4** | Synergy |  |
| *E. coli* A5 | Colistin | 0.24 | **64** | 0.03 | 8 | 0.25 | **8** | **8** | Synergy |  |
| *E. coli* A6 | Colistin | 0.48 | **64** | 0.03 | 8 | 0.19 | **16** | **8** | Synergy |  |
| *E. coli* A7 | Colistin | 0.48 | **32** | 0.03 | 8 | 0.31 | **16** | **4** | Synergy |  |
| *E. coli* A8 | Colistin | 0.24 | **64** | 0.03 | 8 | 0.25 | **8** | **8** | Synergy |  |
| *E. coli* A9 | Colistin | 0.48 | **64** | 0.06 | 8 | 0.25 | **8** | **8** | Synergy |  |
| *E. coli* A10 | Colistin | 0.12 | **64** | 0.03 | 16 | 0.5 | **4** | **4** | Synergy |  |
| *E. coli* A11 | Colistin | 0.32 | **64** | 0.02 | 16 | 0.31 | **16** | **4** | Synergy |  |
| *E. coli* A12 | Colistin | 8.32 | **55** | 0.13 | 8 | 0.16 | **64** | **6.8** | Synergy |  |
| *E. coli* A13 | Colistin | 0.24 | **64** | 0.03 | 16 | 0.38 | **8** | **4** | Synergy |  |
| *E. coli* A15 | Colistin | 0.32 | **64** | 0.02 | 8 | 0.19 | **16** | **8** | Synergy |  |
| *E. coli* A16 | Colistin | 0.48 | **64** | 0.06 | 8 | 0.25 | **8** | **8** | Synergy |  |
| *E. coli* A17 | Colistin | 0.08 | **64** | 0.01 | 8 | 0.25 | **8** | **8** | Synergy |  |
| *E. coli* A18 | Colistin | 1 | **32** | 0.25 | 8 | 0.5 | **4** | **4** | Synergy |  |
| *E. coli* A19 | Colistin | 0.08 | **128** | 0.01 | 16 | 0.25 | **8** | **8** | Synergy |  |
| *E. coli* S2 | Colistin | 0.12 | **32** | 0.03 | 8 | 0.5 | **4** | **4** | Synergy |  |
| *E. coli* S3 | Colistin | 0.12 | **31** | 0.03 | 4 | 0.38 | **4** | **7.75** | Synergy |  |
| *E. coli* S4 | Colistin | 0.24 | **31** | 0.06 | 4 | 0.38 | **4** | **7.75** | Synergy |  |
| *E. coli* S5 | Colistin | 0.24 | **16** | 0.06 | 4 | 0.5 | **4** | **4** | Synergy |  |
| *E. coli* S6 | Colistin | 0.24 | **16** | 0.06 | 4 | 0.5 | **4** | **4** | Synergy |  |
| *E. coli* S7 | Colistin | 0.24 | **16** | 0.06 | 4 | 0.5 | **4** | **4** | Synergy |  |
| *E. coli* S8 | Colistin | 0.24 | **31** | 0.06 | 4 | 0.38 | **4** | **7.75** | Synergy |  |
| *E. coli* S10 | Colistin | 0.24 | **31** | 0.06 | 4 | 0.38 | **4** | **7.75** | Synergy |  |
| *E. coli* S11 | Colistin | 0.24 | **32** | 0.06 | 8 | 0.5 | **4** | **4** | Synergy |  |
| *E. coli* S12 | Colistin | 0.12 | **62** | 0.03 | 8 | 0.38 | **4** | **7.75** | Synergy |  |
| *E. coli* S13 | Colistin | 0.12 | **32** | 0.03 | 8 | 0.5 | **4** | **4** | Synergy |  |
| *E. coli* S14 | Colistin | 0.24 | **62** | 0.06 | 8 | 0.38 | **4** | **7.75** | Synergy |  |
| *E. coli* S15 | Colistin | 0.24 | **16** | 0.06 | 4 | 0.5 | **4** | **4** | Synergy |  |
| *E. coli* S16 | Colistin | 0.24 | **32** | 0.06 | 4 | 0.38 | **4** | **8** | Synergy |  |
| *E. coli* S17 | Colistin | 0.24 | **32** | 0.06 | 4 | 0.38 | **4** | **8** | Synergy |  |
| *E. coli* S18 | Colistin | 0.24 | **32** | 0.06 | 4 | 0.38 | **4** | **8** | Synergy |  |
| *E. coli* S19 | Colistin | 0.24 | **32** | 0.06 | 8 | 0.5 | **4** | **4** | Synergy |  |
| *E. coli* S20 | Colistin | 0.52 | **32** | 0.13 | 8 | 0.5 | **4** | **4** | Synergy |  |
| *E. coli* S21 | Colistin | 0.24 | **8** | 0.06 | 2 | 0.5 | **4** | **4** | Synergy |  |
| *E. coli* S22 | Colistin | 0.24 | **16** | 0.06 | 4 | 0.5 | **4** | **4** | Synergy |  |
| *E. coli* S23 | Colistin | 0.24 | **16** | 0.06 | 4 | 0.5 | **4** | **4** | Synergy |  |
| *E. coli* S24 | Colistin | 0.24 | **16** | 0.06 | 4 | 0.5 | **4** | **4** | Synergy |  |
| *E. coli* S25 | Colistin | 0.52 | **32** | 0.13 | 8 | 0.5 | **4** | **4** | Synergy |  |
| *E. coli* S27 | Colistin | 0.24 | **8** | 0.06 | 2 | 0.5 | **4** | **4** | Synergy |  |
| *K. pneumoniae*  BAA-2470 | Colistin | 1.04 | **64** | 0.13 | 8 | 0.25 | **8** | **8** | Synergy |  |
| *K. pneumoniae*  BAA-2472 | Colistin | 0.48 | **128** | 0.06 | 16 | 0.25 | **8** | **16** | Synergy |  |
| *K. pneumoniae*  BAA-2473 | Colistin | 2.08 | **64** | 0.13 | 8 | 0.19 | **16** | **8** | Synergy | (Hu and Coates., 2021) |
| *K. pneumoniae*  NCTC 13443 | Colistin | 0.48 | **64** | 0.06 | 8 | 0.25 | **8** | **8** | Synergy |  |
| *K. pneumoniae*  A1 | Colistin | 10.24 | **128** | 0.02 | 64 | 0.5 | **512** | **2** | Synergy |  |
| *K. pneumoniae*  A2 | Colistin | 7.68 | **122** | 0.06 | 32 | 0.27 | **128** | **3.8** | Synergy |  |
| *K. pneumoniae*  A3 | Colistin | 2.08 | **126** | 0.13 | 16 | 0.19 | **16** | **7.8** | Synergy |  |
| *K. pneumoniae*  A4 | Colistin | 10.24 | **128** | 0.02 | 32 | 0.25 | **512** | **4** | Synergy |  |
| *K. pneumoniae*  A5 | Colistin | 8.32 | **121** | 0.13 | 32 | 0.28 | **64** | **3.78** | Synergy |  |
| *K. pneumoniae*  A6 | Colistin | 4 | **128** | 0.5 | 16 | 0.25 | **8** | **8** | Synergy |  |
| *K. pneumoniae*  A7 | Colistin | 4.16 | **128** | 0.13 | 32 | 0.28 | **32** | **4** | Synergy |  |
| *K. pneumoniae*  A8 | Colistin | 8.32 | **122** | 0.13 | 32 | 0.28 | **64** | **3.8** | Synergy |  |
| *K. pneumoniae*  A9 | Colistin | 4.16 | **124** | 0.13 | 16 | 0.16 | **32** | **7.75** | Synergy |  |
| *K. pneumoniae*  A10 | Colistin | 3.84 | **131** | 0.06 | 32 | 0.26 | **64** | **4** | Synergy |  |
| *K. pneumoniae*  A11 | Colistin | 10.24 | **128** | 0.02 | 64 | 0.5 | **512** | **4** | Synergy |  |
| *K. pneumoniae*  A12 | Colistin | 4.16 | **124** | 0.13 | 16 | 0.16 | **32** | **7.75** | Synergy |  |
| *K. pneumoniae*  A13 | Colistin | 7.68 | **512** | 0.06 | 128 | 0.26 | **128** | **4** | Synergy |  |
| *K. pneumoniae*  A14 | Colistin | 7.68 | **130** | 0.03 | 32 | 0.25 | **256** | **4** | Synergy |  |
| *K. pneumoniae*  A15 | Colistin | 7.68 | **130** | 0.06 | 32 | 0.27 | **128** | **4** | Synergy |  |
| *K. pneumoniae*  A16 | Colistin | 8.32 | **121** | 0.13 | 32 | 0.28 | **64** | **3.78** | Synergy |  |
| *K. pneumoniae*  A17 | Colistin | 2.56 | **126** | 0.02 | 32 | 0.26 | **128** | **4** | Synergy |  |
| *K. pneumoniae*  A18 | Colistin | 2.56 | **126** | 0.02 | 32 | 0.26 | **128** | **4** | Synergy |  |
| *K. pneumoniae*  A19 | Colistin | 1.04 | **32** | 0.13 | 8 | 0.38 | **8** | **4** | Synergy |  |
| *K. pneumoniae*  A20 | Colistin | 1.92 | **127** | 0.03 | 32 | 0.27 | **64** | **4** | Synergy |  |
| *K. pneumoniae*  A21 | Colistin | 2.56 | **128** | 0.02 | 32 | 0.26 | **128** | **4** | Synergy |  |
| *K. pneumoniae*  A22 | Colistin | 2 | **32** | 0.25 | 16 | 0.13 | **8** | **2** | Synergy |  |
| *K. pneumoniae*  A23 | Colistin | 2.08 | **126** | 0.13 | 16 | 0.19 | **16** | **7.87** | Synergy |  |
| *K. pneumoniae*  A24 | Colistin | 1.92 | **124** | 0.06 | 16 | 0.16 | **32** | **7.75** | Synergy |  |
| *K. pneumoniae*  S1 | Colistin | 1 | **64** | 0.25 | 2 | 0.28 | **4** | **32** | Synergy |  |
| *K. pneumoniae*  S2 | Colistin | 1 | **50** | 0.25 | 1 | 0.27 | **4** | **50** | Synergy |  |
| *K. pneumoniae*  S3 | Colistin | 7.68 | **64** | 0.03 | 32 | 0.5 | **256** | **2** | Synergy |  |
| *K. pneumoniae*  S4 | Colistin | 0.52 | **62** | 0.13 | 8 | 0.38 | **4** | **7.75** | Synergy |  |
| *K. pneumoniae*  S5 | Colistin | 0.12 | **32** | 0.03 | 8 | 0.5 | **4** | **4** | Synergy |  |
| *K. pneumoniae*  S6 | Colistin | 2 | **64** | 0.5 | 16 | 0.5 | **4** | **4** | Synergy |  |
| *K. pneumoniae*  S7 | Colistin | 0.24 | **62** | 0.06 | 8 | 0.38 | **4** | **7.75** | Synergy |  |
| *K. pneumoniae*  S8 | Colistin | 0.24 | **62** | 0.06 | 8 | 0.38 | **4** | **7.75** | Synergy |  |
| *K. pneumoniae*  S9 | Colistin | 0.24 | **64** | 0.06 | 2 | 0.28 | **4** | **32** | Synergy |  |
| *K. pneumoniae*  S10 | Colistin | 2 | **64** | 0.5 | 16 | 0.5 | **4** | **4** | Synergy |  |
| *K. pneumoniae*  S12 | Colistin | 0.52 | **64** | 0.13 | 1 | 0.26 | **4** | **64** | Synergy |  |
| *K. pneumoniae*  S13 | Colistin | 0.12 | **32** | 0.03 | 8 | 0.5 | **4** | **4** | Synergy |  |
| *K. pneumoniae*  S14 | Colistin | 2 | **64** | 0.5 | 2 | 0.28 | **4** | **32** | Synergy |  |
| *K. pneumoniae*  S15 | Colistin | 0.24 | **64** | 0.06 | 16 | 0.5 | **4** | **4** | Synergy |  |
| *K. pneumoniae*  S16 | Colistin | 0.52 | **64** | 0.13 | 16 | 0.5 | **4** | **4** | Synergy |  |
| *K. pneumoniae*  S17 | Colistin | 0.52 | **64** | 0.13 | 16 | 0.5 | **4** | **4** | Synergy |  |
| *K. pneumoniae*  S18 | Colistin | 2 | **64** | 0.5 | 16 | 0.5 | **4** | **4** | Synergy |  |
| *K. pneumoniae*  S19 | Colistin | 0.24 | **32** | 0.03 | 8 | 0.38 | **8** | **4** | Synergy |  |
| *K. pneumoniae*  S20 | Colistin | 2 | **32** | 0.5 | 8 | 0.5 | **4** | **4** | Synergy |  |
| *K. pneumoniae*  S21 | Colistin | 0.52 | **62** | 0.13 | 8 | 0.38 | **4** | **7.75** | Synergy |  |
| *K. pneumoniae*  S22 | Colistin | 0.52 | **16** | 0.13 | 4 | 0.5 | **4** | **4** | Synergy |  |
| *K. pneumoniae*  S23 | Colistin | 2 | **32** | 0.5 | 8 | 0.5 | **4** | **4** | Synergy |  |
| MRSA 252 | Oxacillin | 512 | 16 | 128 | 4 | 0.5 | **4** | **4** | Synergy | (Podoll et al., 2021) |
| MRSA ATCC 33592 | Oxacillin | 128 | 16 | 32 | 4 | 0.5 | **4** | **4** | Synergy |  |
| MSSA ATCC 25923 | Oxacillin | 0.25 | 16 | 0.06 | 4 | 0.5 | **4** | **4** | Synergy |  |
| MSSA NCTC 8325 | Oxacillin | 0.25 | 16 | 0.06 | 4 | 0.5 | **4** | **4** | Synergy |  |
| *M. tuberculosis*  H37Rv | INH | 0.2 | 12.5 | 0.1 | 0.1 | 0.5 | **2** | **125** | Synergy | (Dos Santos et al., 2021) |
|  | PYR | 100 | 12.5 | 3.12 | 3.12 | 0.3 | **32** | **4** | Synergy |  |
| *M. tuberculosis*  T3609 | OFX | 1.25 | 12.5 | 0.62 | 0.62 | 0.5 | **2** | **20** | Synergy |  |
|  | INH | 0.5 | 12.5 | 0.015 | 0.015 | 0.03 | **33** | **833** | Synergy |  |
|  | GAT | 0.62 | 12.5 | 0.31 | 0.31 | 0.5 | **2** | **40** | Synergy |  |
|  | MOX | 1.25 | 12.5 | 0.62 | 0.62 | 0.5 | **2** | **20** | Synergy |  |
|  | SPR | 1.25 | 12.5 | 0.62 | 0.62 | 0.5 | **2** | **20** | Synergy |  |
| *M. tuberculosis*  T113 | CPX | 0.62 | 12.5 | 0.31 | 0.31 | 0.5 | **2** | **40** | Synergy |  |
|  | LVX | 0.62 | 12.5 | 0.31 | 0.31 | 0.5 | **2** | **40** | Synergy |  |
|  | OFX | 1.25 | 12.5 | 0.62 | 0.62 | 0.5 | **2** | **20** | Synergy |  |

Note: The general font is directly compared to the tabular information in the references, and the bold font is calculated according to the explanations in the literature. Abbreviations: MIC, Minimum Inhibitory Concentrations; MFL, mefloquine; INH, isoniazid; PYR, pyrazinamide; OFX, ofloxacin; GAT, gatifloxacin; MOX, moxifloxacin; SPR, sparfloxacin; CPX, ciprofloxacin; LVX, levofloxacin; *P. aeruginosa, Pseudomonas aeruginosa; E. coli, Escherichia coli; K. pneumoniae, Klebsiella pneumoniae;* MRSA, methicillin-resistant *Staphylococcus aureus;* MSSA, methicillin-susceptible *Staphylococcus aureus; M. tuberculosis, Mycobacterium tuberculosis.*

**References**

Dos Santos, M.C., Scaini, J.L.R., Lopes, M.V.C., Rodrigues, B.G., Silva, N.O., Borges, C.R.L., et al., (2021). Mefloquine synergism with anti-tuberculosis drugs and correlation to membrane effects: Biologic, spectroscopic and molecular dynamics simulations studies. *Bioorg Chem* 110, 104786. doi: 10.1016/j.bioorg.2021.104786.

Hu, Y., Coates, A., (2021). Mefloquine enhances the activity of colistin against antibiotic-resistant Enterobacterales in vitro and in an in vivo animal study. Int J Antimicrob Agents 57, 106309. doi: 10.1016/j.ijantimicag.2021.106309.

Kucharski, D.J., Jaszczak, M.K., Boratyński, P.J., (2022). A Review of Modifications of Quinoline Antimalarials: Mefloquine and (hydroxy)Chloroquine. Molecules 27, 1003. doi: 10.3390/molecules27031003.

Podoll, J., Olson, J., Wang, W., Wang, X., (2021). A Cell-Free Screen for Bacterial Membrane Disruptors Identifies Mefloquine as a Novel Antibiotic Adjuvant. Antibiotics (Basel) 10, 315. doi: 10.3390/antibiotics10030315.
